# Supplementary material for: Identification of Adipocytokine Pathway-Related Genes in Epilepsy and Its Effect on the Peripheral Immune Landscape
Source: Brain Sci. 2022 Aug 30;12(9):1156. doi: 10.3390/brainsci12091156 (PMC9497159; doi:10.3390/brainsci12091156)
Supplement: Supplementary file 1 [file brainsci-12-01156-s001.zip › Figure S1.pdf]

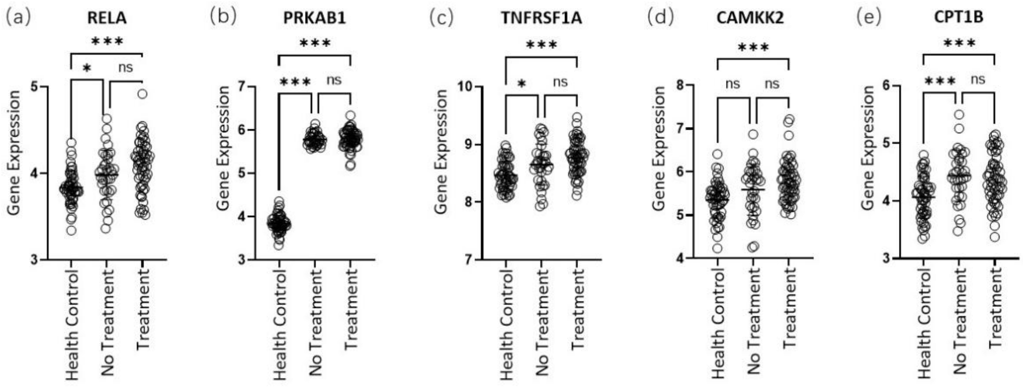

Figure S1. The effect of antiepileptic drugs on the five adipocytokine pathway-related genes. * $p < 0.05$; *** $p < 0.001$; ns, $p > 0.05$.
